# Editorial: Mitochondrial bioenergetics impairments in genetic and metabolic diseases

**DOI:** 10.3389/fphys.2023.1228926

**Published:** 2023-06-02

**Authors:** Christian Bergamini, Elena Bonora, Noah Moruzzi

**Affiliations:** ^1^ Department of Pharmacy and Biotechnology, University of Bologna, Bologna, Emilia-Romagna, Italy; ^2^ Department of Medical and Surgical Sciences, University of Bologna, Bologna, Italy; ^3^ Department of Molecular Medicine and Surgery, Karolinska Institutet (KI), Stockholm, Sweden

**Keywords:** mitochondria, bioenergetics, metabolic impairment, inherited human disorders, mitochondrial dysfunction

Mitochondria are represented as the powerhouse of the cells providing energy through a complex series of bioenergetic reactions, as they play pivotal roles in the catabolism of sugars, lipids, and amino acids. Moreover, they are emerging as signaling organelles by maintaining cellular redox homeostasis, generating biosynthetic precursors for macromolecules, and orchestrating processes such as autophagy and apoptosis (Turrens, 2003; Spinelli and Haigis, 2018; Suomalainen and Battersby, 2018). The considerable resources that an individual cell must provide to maintain mitochondrial integrity and thus their functions underscore their essential role and provide a rationale for their involvement in many pathologies. Over the years, mitochondrial dysfunctions have been increasingly associated with inherited human disorders and linked to diseases characterized by metabolic imbalances, such as metabolic syndrome, diabetes, and obesity.

Our cells house hundreds to thousands of mitochondria, each with 2–10 copies of mitochondrial DNA (mt-DNA) in a network of fusing and budding organelles. Mitochondrial diseases are genetically inherited and characterized by mutations harbored in both the circular 16,569 bp mitochondrial DNA (mtDNA) and the nuclear DNA (nDNA), which are jointly responsible for the transcription of mitochondrial proteins. In fact, the mtDNA includes only 37 genes, including 22 tRNAs and 2 rRNAs essential for mtDNA-specific translation of the 13 encoded respiratory chain subunits (Stenton and Prokisch, 2020). In the nDNA, over thousand mitochondrial localizing proteins are encoded, translated in the cytoplasm, and translocated to the mitochondria by an elaborate protein import machinery. Mitochondrial diseases and their outcome depend on which gene is affected and lead to a wide variety of phenotypes, ranging from disruption of the oxidative phosphorylation (OXPHOS) activity to impairment of integral mitochondrial functions. Since the first report by the father of mitochondrial medicine Rolf Luft in the ´60, many diseases originating by primary mitochondrial defects have emerged (Luft et al, 1962).

One of the most studied mutation is the m.3243A>G mutation in the mtDNA, which has been associated with different mitochondrial diseases. This change causes an erroneous translation of a component of the mitochondrial tRNA leading to mitochondrial impairment. In a review published in this Research Topic, Li et al (2022) summarized genetic and phenotypical features of this specific mutation. Moreover, they highlight disease-modify genetic interventions, which are becoming pivotal during the last 10 years in the clinical field.

Gene haploinsufficiency, a condition where only one copy of a gene is functional, can also result in mitochondrial diseases. This is displayed in this Research Topic by Hernández-Camacho et al. regarding the nDNA encoded protein ADCK2 (Bellusci et al, 2021). ADCK2 is a member of the aarF domain-containing mitochondrial protein kinase family and its haploinsufficiency results in coenzyme Q_10_ deficiency in skeletal muscle, therefore impairing beta-oxidation and physical performance (Navas et al, 2021). Additionally, the decreased production of coenzyme Q_10_ may also determine increased cellular oxidative stress, which can further exacerbate mitochondrial dysfunctions and contribute to the development of disease. In their original article, Hernández-Camacho et al. reported that caloric restriction rescue aerobic metabolism and differentiation capacity of skeletal muscle in mice caused by ADCK2 haploinsufficiency. They showed an increased coenzyme Q_10_ levels and oxygen consumption rate, based on both glucose and fatty acids substrates in mitochondria isolated from skeletal muscle, along with increased mitochondrial mass. Finally, they proposed that caloric restriction should be considered an alternative for the treatment of mitochondrial diseases, and particularly for those with a mild pathology caused by nuclear genome defects.

As metabolic powerhouse of the cell but also as main origin of intracellular reactive oxygen species, it is not surprising that mitochondria link metabolism with cancer exacerbation and progression. Especially, the Warburg effect, i.e., the metabolic switch of ATP production between aerobic glycolysis and OXPHOS, has been long described and characterized in most cancerous cells, allowing them to grow and adapt to a poor oxygen supplied environment (Liberti and Locasale, 2016). In this Research Topic, Freire Jorge et al. discuss the naked mole-rats and the concomitantly long cancer-free lives despite their small size and high metabolic rate. Their unique metabolism that suits their underground habitat, and the ability to tolerate and exercise in hypoxic environments without significant acidosis, is in some way related to their remarkable resistance to cancer. Thus, they hypothesize that the low cancer incidence in these rats may be related to a hard-wired coupling of glycolysis with oxidative phosphorylation, leading to the inability to display a Warburg effect. By relying on oxidative phosphorylation for energy production rather than glycolysis, naked mole-rat cells may be less susceptible to the accumulation of mutations and other genetic changes that can lead to cancer. Emerging in the field of cancer as well as neurodegeneration is the endogenous inhibitor of the ATP synthase (IF1). This protein described across animals and plant species is the subject of the review by Gatto et al. in this Research Topic, where they provide a complete view of the findings in the literature regarding IF1 functions, mechanisms of actions and relationship to diseases with a particular focus on cancer and neurodegeneration.

Impairments in energy metabolism, mitochondrial bioenergetics, and even cancer development are linked to metabolic syndrome, diabetes and obesity. Over the years, evidence that describes alterations in mitochondrial number, morphology, or functions in metabolic syndrome and diabetes has been accumulating. However, whether these mitochondrial changes are the cause or consequence of the diabetic condition is under debate as well as the exact mechanisms behind these changes. Of special interest is the metabolic memory that tissue exposed to hyperglycemia can carry over even when the normoglycemia is re-established. In this context, bile acids have recently emerged as a signaling molecule that can regulate glucose homeostasis and affect metabolic syndrome and diabetes (Rajani and Jia, 2018). In this Research Topic, Zheng et al. address the bile acid recirculation and the effect on glucose metabolism after proximal small bowel bypass, a surgical procedure that involves rerouting a portion of the small intestine to reduce the absorption of nutrients, including glucose. This procedure has been shown to improve glucose tolerance and reduce insulin resistance in both animal models and human patients with type 2 diabetes. Previously, they designed a side-to-side jejunoileal bypass, which bypasses the proximal small intestine and can significantly improve glucose metabolism with elevated total serum bile acid levels (Duan et al, 2015). Here, using the diabetic type 2 model Goto-Kakizaki (GK) rat model, they investigated the mechanism of elevated bile acid and its role in glucose regulation.

Overall, impaired mitochondrial bioenergetics is a common feature of many genetic and metabolic diseases. Understanding the underlying mechanisms of mitochondrial dysfunctions in these diseases may provide new therapeutic targets for their treatment, especially in this era of genetic manipulation approaches which is revolutionizing personalized target medicine.

In this Research Topic, we sought to collect experimental studies supporting descriptions of defined mechanisms and physiological responses underlying metabolic diseases, together with studies of phenotypic features of gene mutations directly or indirectly affecting mitochondrial bioenergetics or connected processes. In our opinion, these types of experimental studies are a pivotal step necessary to generate innovative and personalized therapeutic strategies.
